# Integrating salpingoscopy and immunohistochemistry to improve tubal infertility diagnosis: a retrospective cohort study

**DOI:** 10.3389/frph.2025.1701315

**Published:** 2025-11-27

**Authors:** Adalyat Alenova, Anuar Korkan, Almagul Kauysheva, Islam Magalov

**Affiliations:** 1 Kazakhstan Medical University «KSPH», Almaty, Kazakhstan; 2 Medical Center RAHAT, Almaty, Kazakhstan; 3 Department of Obstetrics and Gynecology № 1, Medical University of Astana, Astana, Kazakhstan; 4 Department of Science and Human Resources, Ministry of Health of the Republic of Kazakhstan, Astana, Kazakhstan; 5 Department of Obstetrics and Gynecology 1, Azerbaijan Medical University, Baku, Azerbaijan; 6 Department of Obstetrics and Gynecology, Baku Branch of the First Moscow State Medical University, Baku, Azerbaijan

**Keywords:** infertility, diagnostic methods, salpingoscopy, immunohistochemistry, reproductive technologies, fallopian tubes

## Abstract

**Introduction:**

Tubal factor infertility is one of the most prevalent and diagnostically complex causes of female infertility. Standard imaging modalities, including hysterosalpingography and laparoscopy, frequently fail to detect subtle mucosal or molecular abnormalities. This study investigated whether combining salpingoscopy with immunohistochemical (IHC) profiling improves diagnostic precision and prognostic assessment in women with tubal-factor infertility.

**Methods:**

This retrospective observational cohort study included 197 women who underwent laparoscopy between April 2020 and February 2025. Salpingoscopy was used to grade mucosal pathology severity, and ampullary biopsies were analyzed by IHC for CD138, Ki-67, and estrogen/progesterone receptors. A composite prognosis score incorporated clinical history, duration of conservative therapy, salpingoscopic findings, and IHC results. Spearman's rank correlation assessed associations between diagnostic parameters and pregnancy outcomes (natural or assisted).

**Results:**

Higher salpingoscopic severity scores were significantly associated with reduced pregnancy likelihood (*r* = −0.457; *p* < 0.0001). Elevated IHC scores similarly correlated with decreased conception rates (*r* = −0.263; *p* = 0.0002). Tubal dilations and adhesions showed negative associations with reproductive outcomes (*r* = −0.232; *p* = 0.001). In contrast, the composite prognosis score demonstrated a strong positive correlation with treatment success (*r* = 0.578; *p* < 0.0001).

**Discussion:**

Integrating salpingoscopic visualization with IHC profiling enhances detection of subtle mucosal and molecular abnormalities that are frequently missed by conventional diagnostic approaches. This combined modality offers superior prognostic accuracy and may guide personalized surgical and assisted reproductive treatment strategies. Ethical approval was obtained from the Ethics Committee of the Kazakhstan Medical University «KSPH» (IRB-64-2023).

## 1 Introduction

Infertility affects an estimated 12.6%–17.5% of couples of reproductive age worldwide and is a growing public health issue with complex psychological and socioeconomic consequences (1). Among the causes of female infertility, tubal factor infertility is one of the most prevalent and diagnostically challenging etiologies (2). It accounts for 30%–40% of all women's infertility cases (3). Tubal factor infertility can result from obstruction, scarring, or impaired function of the fallopian tubes (4). The routine assessment of tubal patency typically involves the use of hysterosalpingography (HSG), sonohysterography (SHG), and hysterosalpingo-contrast sonography (HyCoSy). These imaging methods are accessible and provide non-invasive detection of anatomical blockages (5, 6). However, they do not provide information on subtle epithelial or functional abnormalities (7). Even laparoscopy, currently considered the gold standard for evaluating tubal and pelvic pathology, lacks the resolution to identify mucosal damage, ciliary dysfunction, or subclinical inflammation (8). These limitations may account for cases of unexplained infertility and reduce the effectiveness of both natural and assisted reproductive technology (ART) treatments (9, 10).

In clinical practice among underrepresented and resource-constrained populations, such as in Central Asia, such diagnostic limitations are particularly evident (11). In Kazakhstan, female infertility accounts for the majority of reported infertility cases, but many patients undergo empirical treatment without a definitive diagnosis (12, 13). This further highlights the need for improved diagnostic strategies capable of assessing not only tubal patency but also functional and molecular integrity. However, few studies have systematically assessed the combined diagnostic and prognostic value of salpingoscopy and immunohistochemistry (IHC) in tubal factor infertility. While each method has shown promise separately (3, 14), their integration into routine evaluation remains limited in both clinical practice and the literature.

The combination of salpingoscopy and IHC analysis may offer a novel opportunity to bridge this diagnostic gap. Salpingoscopy is an endoscopic technique that allows for the direct visualization of the inside of the fallopian tube (14). Recent studies have indicated that specific salpingoscopic findings, such as loss of mucosal folds or intraluminal adhesions, are associated with reduced fertility potential and poor ART outcomes (15–17). IHC analysis allows for molecular profiling of the tubal epithelium, including markers of inflammation (e.g., CD138), hormone receptor status (e.g., progesterone and estrogen), and cell proliferation (e.g., Ki-67 and p53) (18, 19). Increased expression of CD138+ plasma cells and elevated Ki-67 indices in the tubal epithelium have been linked to chronic inflammation and ciliary dysfunction (20, 21).

By integrating anatomical and cellular-level assessment, these methods may increase diagnostic precision, leading to targeted therapeutic strategies and enhancing reproductive outcomes.

This observational retrospective study aimed to evaluate whether combining salpingoscopy and IHC profiling improves diagnostic precision and better predicts pregnancy outcomes compared to conventional assessment methods. We further investigated whether these diagnostic indicators independently predict treatment success, and whether they can identify patients with poorer reproductive prognosis who may benefit from earlier ART or targeted interventions. We hypothesized that the integration of salpingoscopic grading and IHC profiling would offer prognostic value beyond traditional assessment and support more personalized fertility treatment planning.

## 2 Methods

### 2.1 Study population and study design

**Figure 1:**
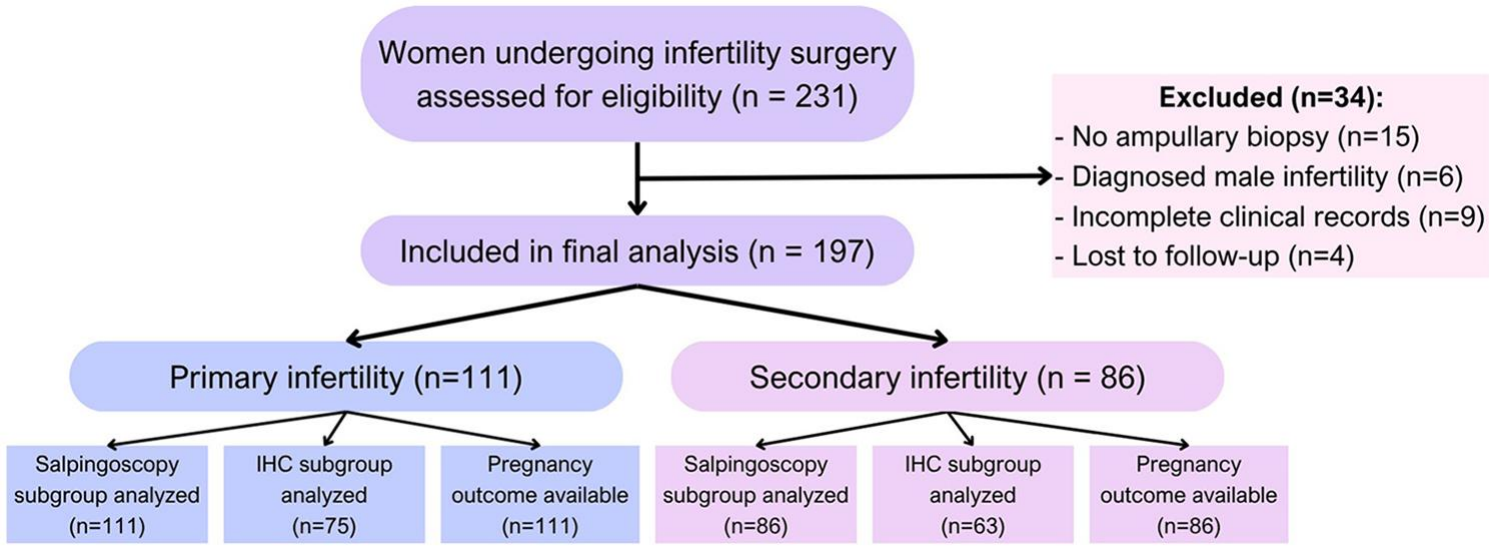
Flow diagram of the selection of the participants.

This observational retrospective cohort study included 197 women participants from collaborating reproductive health centers in Kazakhstan between April 2020 and February 2025. The sample size was determined by enrolling all eligible cases between April 2020 and February 2025 in collaborating centers. Inclusion criteria were women aged 18–45 years with a clinical diagnosis of primary or secondary infertility, defined as failure to conceive after 12 months of regular unprotected intercourse. All the patients had an indication for diagnostic laparoscopy based on unexplained infertility, suspected tubal factor, or failed ART attempts. The exclusion criteria included patients with incomplete medical records, absence of ampullary biopsy, loss to follow-up within 12 months, or evidence of male-factor infertility as the sole diagnosis (Figure 1). Patients with missing or incomplete key clinical data, such as ampullary biopsy results or follow-up pregnancy outcomes, were also excluded from the analysis. No data imputation was performed. Selection bias was minimized by the inclusion of multiple centers and protocolized surgical approaches.

The participants were categorized into two groups, namely primary infertility (*n* = 111), with no prior pregnancies, and secondary infertility (*n* = 86), with at least one prior pregnancy but failure to conceive again.

### 2.2 Ethical consideration

The study was approved by the local ethics committee (IRB-64-2023). All the participants provided written informed consent before inclusion. The study was conducted according to the Declaration of Helsinki. All data were anonymized before the analysis to protect patient confidentiality, and the study complied with relevant data protection regulations.

### 2.3 Diagnostic procedures

During the laparoscopies, salpingoscopies were performed using a microendoscope. Tubal patency was assessed by chromopertubation. Mucosal appearance, adhesion, stenosis, and dilation were systematically documented. Mucosal appearance was graded based on the standardized “F-score” system (22), assigning one point for each of six features (adhesions, loss of folds, rounded fold edges, debris, foreign bodies, and abnormal vessels) observed during salpingoscopy. Each abnormal finding resulted in the addition of 1 point to the F-score, and the maximum was 12 points.

Tissue biopsies from the ampullary region were collected for IHC analysis. The biopsies were taken from the epithelial lining of the ampullary portion of the fallopian tube, mainly from the mucosal surface and occasionally closer to the fimbrial end. The most visibly altered epithelial areas were preferentially sampled. Smooth muscle tissue was not collected, as the fallopian tube wall is thin, prone to bleeding, and considered non-informative for the intended analyses.

Immunohistochemical staining was performed in the affiliated clinical pathology laboratory according to their standard diagnostic protocols; the authors did not perform the IHC procedures themselves. For each patient, the finalized pathology reports and slide assessments were provided to the investigators and reviewed for this study. The standard panels included the expression of various protein markers, such as Ki-67, p53 (23), estrogen and progesterone receptors (24), and CD138 (25), which indicate cell proliferation, inflammatory processes, or the presence of precancerous changes.

Existing pathology reports and slide assessments were reviewed and categorized based on marker expression patterns. The results were grouped into three categories for analysis: (1) IHC not performed, with histologically normal epithelium; (2) positive expression, indicating abnormal findings such as CD138+ plasma cells, elevated Ki-67 index, or aberrant hormone receptor profiles; and (3) negative expression, defined as the absence of pathological immunoreactivity. Discrepancies between reviewers were resolved by consensus.

Pregnancy outcomes were identified retrospectively through medical record review of the 12 months following surgery. A pregnancy was considered confirmed if documented as either a biochemical pregnancy (positive β-hCG) or a clinical pregnancy (ultrasound-confirmed intrauterine gestation), achieved either through natural conception or ART. Treatment success was defined as any documented pregnancy within the follow-up period.

### 2.4 Data collection

Clinical data were retrospectively collected from medical records and included patient age, type of infertility (primary or secondary), gynecological comorbidities, history of STIs, spermogram results of male partners, and morphological findings noted during salpingoscopy. Two coordinators independently abstracted key variables, and a third reviewer resolved any discrepancies.

### 2.5 Statistical analysis

The statistical analyses were performed using GraphPad Prism 10.4.2. Continuous variables are reported as medians with interquartile ranges (IQRs). Group comparisons were performed using the chi-square (*χ*^2^) test. Spearman's rank correlation was used to explore the relationship between the tubal pathology features and pregnancy outcome. A *p*-value < 0.05 was considered statistically significant.

### 2.6 Reporting guidelines

This study adhered to the STROBE (Strengthening the Reporting of Observational Studies in Epidemiology) guidelines. A completed checklist is provided in the Supplementary Materials.

## 3 Results

### 3.1 Age distribution

The age range of the participants was 23–43 years (*n* = 197). The IQR was 28–37 years, with a median age of 31 years. The 25th percentile was 28 years, and the 75th percentile was 37 years, while the minimum and maximum ages were 23 and 43 years, respectively. Thus, 50% of the participants were between 28 and 37 years old. The distribution appears symmetrical, as the median (31 years) is close to the midpoint of the IQR. The study participants predominantly belonged to the young and middle-aged groups, which may reflect the characteristics of the study or the target population.

### 3.2 Clinical characteristics

**Figure 2:**
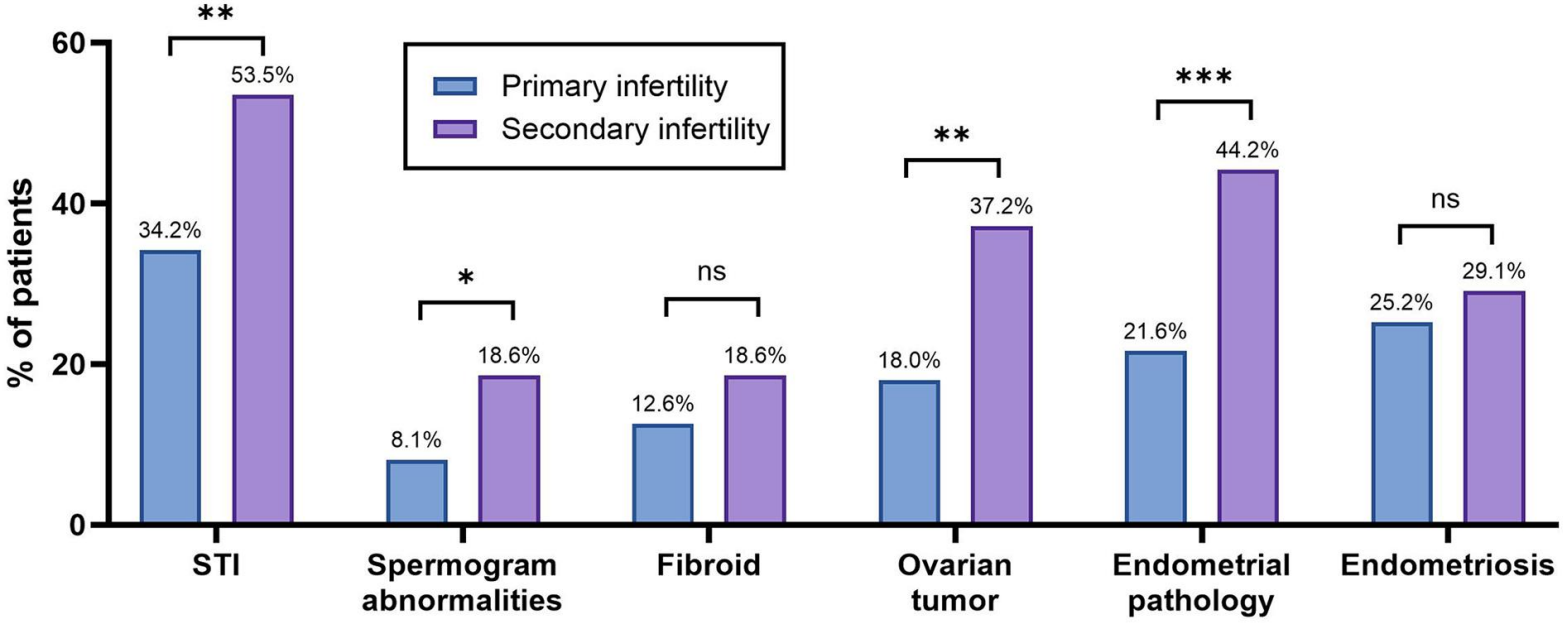
Clinical characteristics of the patients with primary or secondary infertility. The bar graph shows the percentage of patients with specific conditions across infertility types. Statistical significance was identified using the chi-square (*χ*^2^) test, with * denoting *p* < 0.05, ** denoting 0.001 < *p* < 0.01, *** denoting 0.0001 < *p* < 0.001, **** denoting *p* < 0.0001, and ns denoting no statistically significant difference.

Participants were categorized into two groups, namely, primary infertility (*n* = 111) and secondary infertility (*n* = 86). Clinical data were collected retrospectively from the medical records of the patients, including comorbid gynecological diseases, the presence of STIs, and spermogram abnormalities in their male partners (Table 1). These clinical characteristics were then compared between the two groups to determine any significant differences (Figure 2).

**Table 1:** Clinical characteristics of the patients with primary or secondary infertility.

Clinical characteristics	Primary infertility, *n* (%)	Secondary infertility, *n* (%)
STI	38 (34.2)	46 (53.5)
Spermogram abnormalities	9 (8.1)	16 (18.6)
Fibroid	14 (12.6)	16 (18.6)
Ovarian tumor	20 (18.0)	32 (37.2)
Endometrial pathology	24 (21.6)	38 (44.2)
Endometriosis	28 (25.2)	25 (29.1)

The prevalence of ovarian tumors and endometrial pathologies was statistically significantly higher (*p* = 0.0024 and *p* = 0.0007, respectively) among the patients with secondary infertility compared to those with primary infertility. In the primary infertility group, STIs were identified in 34.2% (*n* = 38), while in the secondary infertility group, STIs were identified in 53.5% (*n* = 46). The difference was found to be statistically significant (*p* = 0.0067). Male factor infertility was also analyzed, as spermogram abnormalities were found in 8.1% and 18.6% of the male partners in the primary and secondary infertility groups, respectively. This was significantly higher in the secondary infertility group (*p* = 0.0282).

### 3.3 Tubal patency

**Figure 3:**
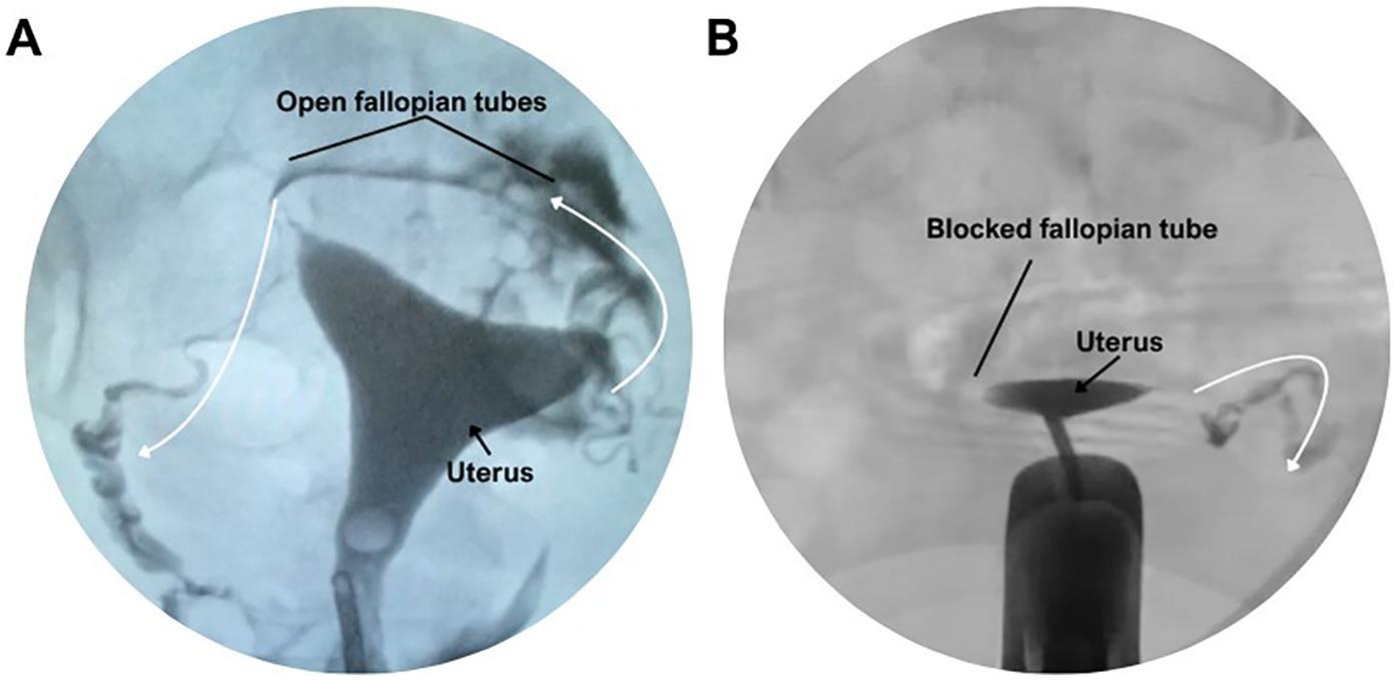
Hysterosalpingographic evaluation of fallopian tube patency. **(A)** Normal hysterosalpingogram showing bilateral contrast passage through open fallopian tubes. **(B)** Hysterosalpingogram demonstrating a blocked fallopian tube with no contrast spill.

**Figure 4:**
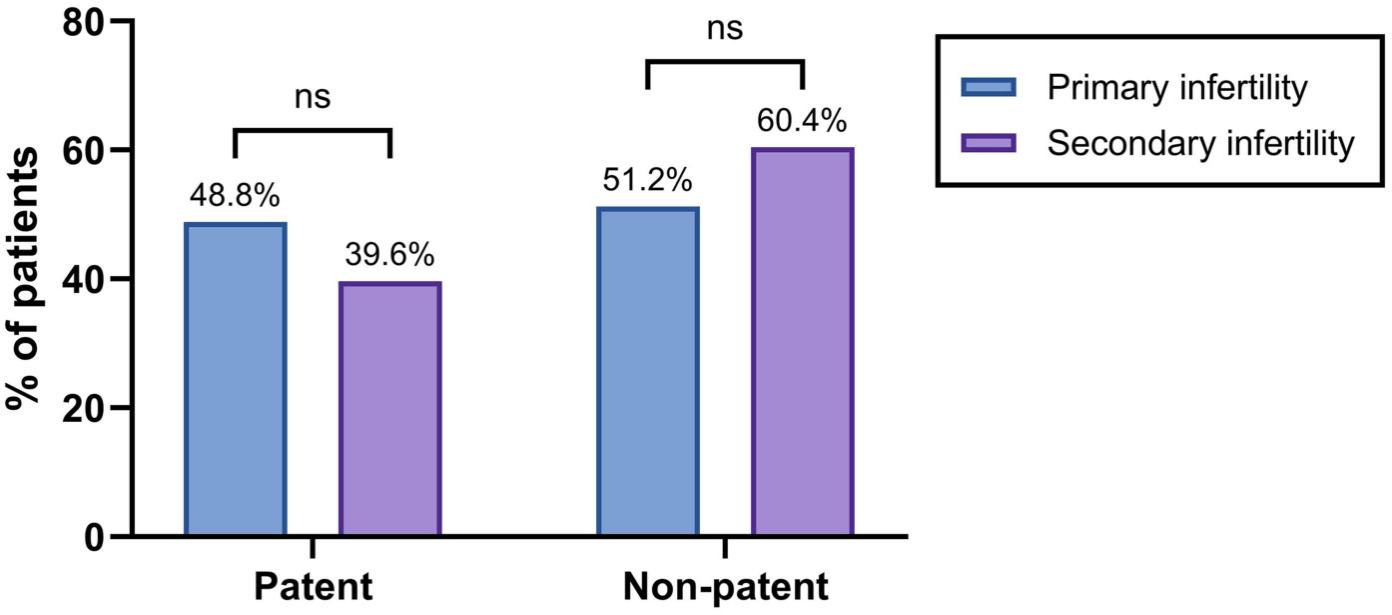
Tubal patency in the infertility groups. The bar graph shows the percentage of patients with patent or non-patent fallopian tubes based on hysterosalpingography. Statistical significance was identified using the chi-square (*χ*^2^) test, with * denoting *p* < 0.05, ** denoting 0.001 < *p* < 0.01, *** denoting 0.0001 < *p* < 0.001, **** denoting *p* < 0.0001, and ns denoting no statistically significant difference.

Hysterosalpingography was performed in 197 patients for diagnostic assessment of tubal patency (Figure 3). Bilateral contrast passage, indicating open fallopian tubes, was observed in 48.8% of the women with primary infertility and in 39.6% of those with secondary infertility. The remaining patients exhibited unilateral or bilateral tubal obstruction. However, the statistical analysis revealed no significant difference between the two groups (Figure 4).

### 3.4 Duration of conservative infertility treatment

Among 197 patients, a total of 51.3% (*n* = 101) underwent conservative therapy for less than 5 years, while 48.7% (*n* = 96) received treatment for more than 5 years. In the primary infertility group, 53.2% (*n* = 59) had conservative infertility treatment for more than 5 years, with 43.0% (*n* = 37) in the secondary infertility group receiving the same. However, the statistical analysis showed no significant difference between the two groups (Supplementary Figure S1).

### 3.5 Immunohistochemical analysis

A positive IHC result indicated abnormal findings, such as the presence of CD138-positive plasma cells, an elevated Ki-67 proliferation index, p53 overexpression, or aberrant estrogen and progesterone receptor profiles, reflecting inflammatory or precancerous alterations in the tubal epithelium. A negative result denoted the absence of pathological immunoreactivity, with normal marker expression consistent with histologically healthy tissue.

**Figure 5:**
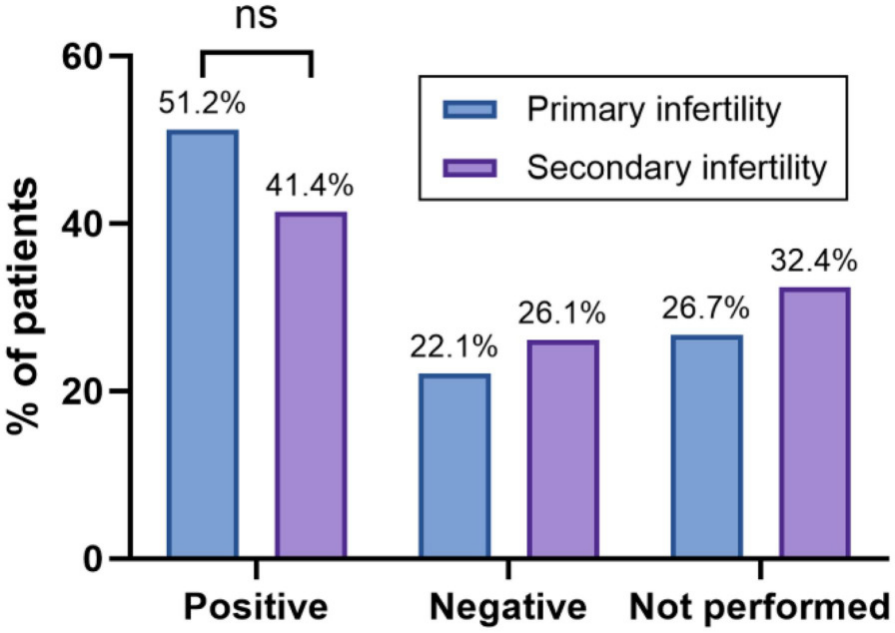
The bar graph shows the percentage of patients with positive, negative, or unperformed IHC findings, stratified by infertility type. Statistical significance was identified using the chi-square (*χ*^2^) test, with * denoting *p* < 0.05, ** denoting 0.001 < *p* < 0.01, *** denoting 0.0001 < *p* < 0.001, **** denoting *p* < 0.0001, and ns denoting no statistically significant difference.

According to the results of the IHC profiling, 51.2% of the patients with primary infertility had a positive result, 22.1% had a negative result, and 26.7% did not undergo the assessment due to having a completely healthy tube. Among the patients with secondary infertility, 41.4% had a positive result, 26.1% had a negative result, and 32.4% did not undergo the assessment due to having a completely healthy tube (Figure 5).

### 3.6 Natural conception prognosis

**Figure 6:**
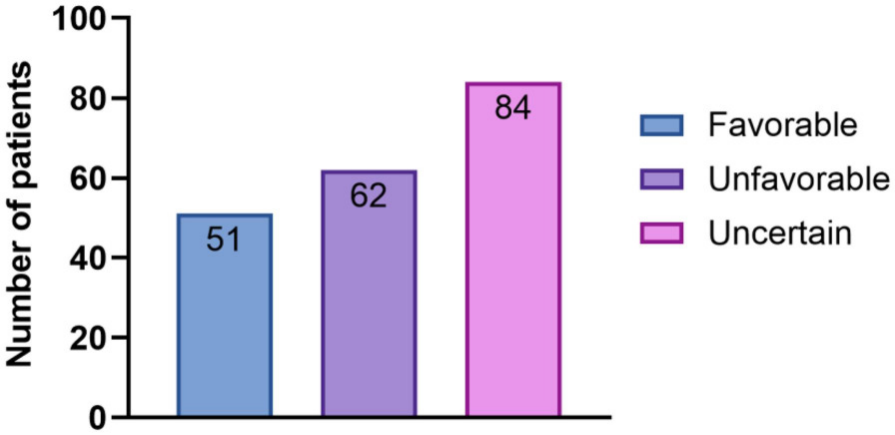
The bar chart shows the distribution of the prognosis categories for spontaneous pregnancy. A total of 197 patients were categorized as having a favorable (*n* = 51), unfavorable (*n* = 62), or uncertain (*n* = 84) prognosis for natural conception, based on clinical and diagnostic criteria.

Considering the patients’ medical history, results of conservative treatment, and examination findings from IHC and salpingoscopy, a prognosis score (from 0 to 10) for the likelihood of natural conception was generated. Thus, 51 women were assigned a “favorable” prognosis, 62 an “unfavorable” prognosis, and 84 an “uncertain” prognosis (Figure 6). Pregnancy outcomes were reported as not pregnant, ART, or natural conception.

In the “favorable” prognosis group, 21 patients conceived naturally, and 30 patients conceived with the help of ART. In the “unfavorable” prognosis group, two patients conceived naturally, 35 patients conceived with the help of ART, and 25 were unable to become pregnant. In the “uncertain” prognosis group, 30 patients conceived naturally, 49 patients conceived with the help of ART, and eight were unable to become pregnant. In the “favorable” group, the pregnancy rate was 100% (21 natural, 30 ART). In the “unfavorable” group, 60.5% achieved pregnancy (2 natural, 35 ART), while in the “uncertain” group, 94% conceived (30 natural, 49 ART). Overall, 167 of the 197 patients (84.8%) achieved pregnancy within 12 months, including 53 (26.9%) via natural conception and 114 (57.9%) via ART.

A comparison of pregnancy outcomes between the women with primary and secondary infertility revealed no statistically significant differences. While the natural conception and ART pregnancy rates were slightly higher in the secondary infertility group, a chi-square test indicated that these differences were not statistically significant (*p* = 0.111). This suggests that, within this cohort, infertility type alone was not a strong predictor of treatment outcome.

### 3.7 Correlation of diagnostic parameters with pregnancy outcome

The tube severity score was calculated based on salpingoscopic grading (“F-score” system), ranging from normal to severely pathological (from 0 to 12) (22). Spearman's rank correlation analysis (Table 2) showed that higher severity scores in the right fallopian tube were significantly associated with a lower likelihood of achieving pregnancy, both naturally and with ART (*r* = −0.457, *p* < 0.0001). A similar but weaker relationship was observed for the left tube severity score (*r* = −0.243, *p* = 0.0005). The presence of dilations or adhesions is also associated with significantly lower chances of treatment success.

**Table 2:** Spearman's rank correlation analysis of the association between the diagnostic parameters and pregnancy outcome.

Parameter	Spearman’s *r*	*p*-Value
Right tube severity score	−0.457	<0.0001
Left tube severity score	−0.243	0.0005
Dilation	−0.233	0.001
Adhesions	−0.232	0.001
IHC score	−0.263	0.0002
Prognosis score	0.578	<0.0001

The IHC findings also demonstrated a slight negative correlation with pregnancy outcome (*r* = −0.263, *p* = 0.0002). In contrast, the prognosis score showed a strong positive association with pregnancy (*r* = 0.578, *p* < 0.0001), indicating that higher predicted probabilities were related to observed treatment success.

Among all the diagnostic parameters, the right tube severity score demonstrated the strongest negative correlation with pregnancy outcome (*r* = –0.457), while the composite prognosis score was the strongest positive predictor (*r* = 0.578).

## 4 Discussion

In this retrospective cohort study of 197 patients who underwent surgical evaluation for infertility, the integration of salpingoscopy and IHC analysis demonstrated a significant association with treatment outcomes. These diagnostic techniques provided complementary anatomical and molecular information in addition to conventional assessments. To our knowledge, this is one of the first studies to integrate salpingoscopic grading and IHC profiling of the fallopian tube epithelium in a large cohort, and to demonstrate their combined predictive value for fertility outcomes.

It was observed that higher salpingoscopic severity scores in both the right and left fallopian tubes correlated negatively with the likelihood of achieving pregnancy (Spearman’s *r* = −0.457 and −0.243, respectively; *p* < 0.0001 and *p* = 0.0005). Moreover, the presence of tubal dilations and adhesions was linked to poorer reproductive treatment outcomes (*r* = −0.233 and −0.232, both *p* = 0.001), highlighting the prognostic value of detailed mucosal evaluation. Several studies have already indicated the importance of salpingoscopy in fertility prognosis. It was demonstrated that the addition of salpingoscopy to laparoscopy significantly increased the accuracy when predicting short-term pregnancy (15, 26). Our findings mirror this, with higher mucosal severity scores correlating strongly with reduced pregnancy rates.

The negative correlation between IHC score, which reflects markers of inflammation, hormonal receptor status, and proliferation, and treatment outcome (*r* = −0.263, *p* = 0.0002) suggests that subclinical epithelial or inflammatory changes may significantly affect tubal function even when gross patency is maintained. These findings align with other studies that indicated that chronic endometritis and increased CD138^+^ cell counts adversely affect fertility and in vitro fertilization (IVF) treatment outcomes (27, 28). By directly sampling ampullary biopsies, our combined approach detects functional impairment that may be missed by HSG, SHG, or laparoscopy alone.

Importantly, a composite prognosis score, incorporating medical history, conservative therapy duration, and combined diagnostic findings, was calculated. It showed a strong positive association with treatment success (*r* = 0.578, *p* < 0.0001), validating its potential application as a clinical decision-making aid. The patients who were classified according to the composite prognosis score as having a favorable prognosis were more likely to conceive, either naturally or via ART, compared to those with unfavorable or uncertain prognoses.

Although some studies suggest different treatment responses in patients with primary or secondary infertility, particularly in response to ART or expectant management, the evidence remains mixed (29, 30). In our study, we did not observe a statistically significant difference in pregnancy outcomes between the patients with primary and secondary infertility (*p* = 0.111), suggesting that the combined diagnostic approach may be more predictive than infertility type alone.

These results support the need to add salpingoscopic visualization and molecular profiling to standard tubal assessments. Salpingoscopy allows for a high-resolution investigation of mucosal integrity, leading to the discovery of possible stenosis, dilation, and adhesions. IHC provides an understanding of the inflammatory and proliferative processes that influence ciliary function and epithelial health. Combined, these tools permit more accurate diagnoses and better-tailored treatment strategies. In particular, this approach may reduce time to pregnancy by identifying the patients who are unlikely to benefit from further empirical treatment and prioritizing them for ART. It may also help avoid unnecessary surgical interventions in patients with unremarkable mucosal and molecular findings.

Several limitations need to be acknowledged. The retrospective, multicenter design can introduce selection bias, and the cohort predominantly reflects practices in Kazakhstan, which may limit its generalizability. To avoid these limitations, data were collected from multiple large centers that follow standard assessment protocols. In addition, all the participating centers were located in Kazakhstan, and while they followed standardized diagnostic and treatment protocols, generalizability may be limited to similar healthcare settings in Central Asia or countries with comparable reproductive health infrastructure. Another limitation is the potential variability in salpingoscopic image interpretation and IHC scoring across the centers. Although our coordinators and pathologists followed standard protocols, interobserver reproducibility was not formally assessed. Future work should focus on developing training modules and digital tools to support diagnostic consistency. Moreover, future studies should validate these findings in larger, more diverse populations and evaluate the cost-effectiveness of the combined diagnostic protocol. Adding genomic or proteomic biomarkers to this salpingoscopic and IHC protocol may further improve its prognostic potential. A comprehensive multimodal diagnostic protocol could optimize personalized treatment, improve pregnancy rates, and reduce the weaknesses of empirical infertility therapies.

## 5 Conclusions

Integrating salpingoscopy and IHC enhances diagnostic and prognostic accuracy in tubal factor infertility. The proposed composite model reliably identifies the patients who are likely to benefit from surgical repair vs. those who require early ART intervention. Prospective validation of this multimodal approach may further optimize personalized treatment and improve reproductive outcomes.

## Data Availability

The data analyzed in this study are subject to the following licenses/restrictions: the datasets used and/or analyzed in the current study are available from the corresponding author on reasonable request. Requests to access these datasets should be directed to Adalyat Alenova, phd.surgeon.alenova@gmail.com.
