# Supplementary figures and images for: Integrating salpingoscopy and immunohistochemistry to improve tubal infertility diagnosis: a retrospective cohort study
Source: Front Reprod Health. 2025 Nov 27;7:1701315. doi: 10.3389/frph.2025.1701315 (PMC12696168; doi:10.3389/frph.2025.1701315)

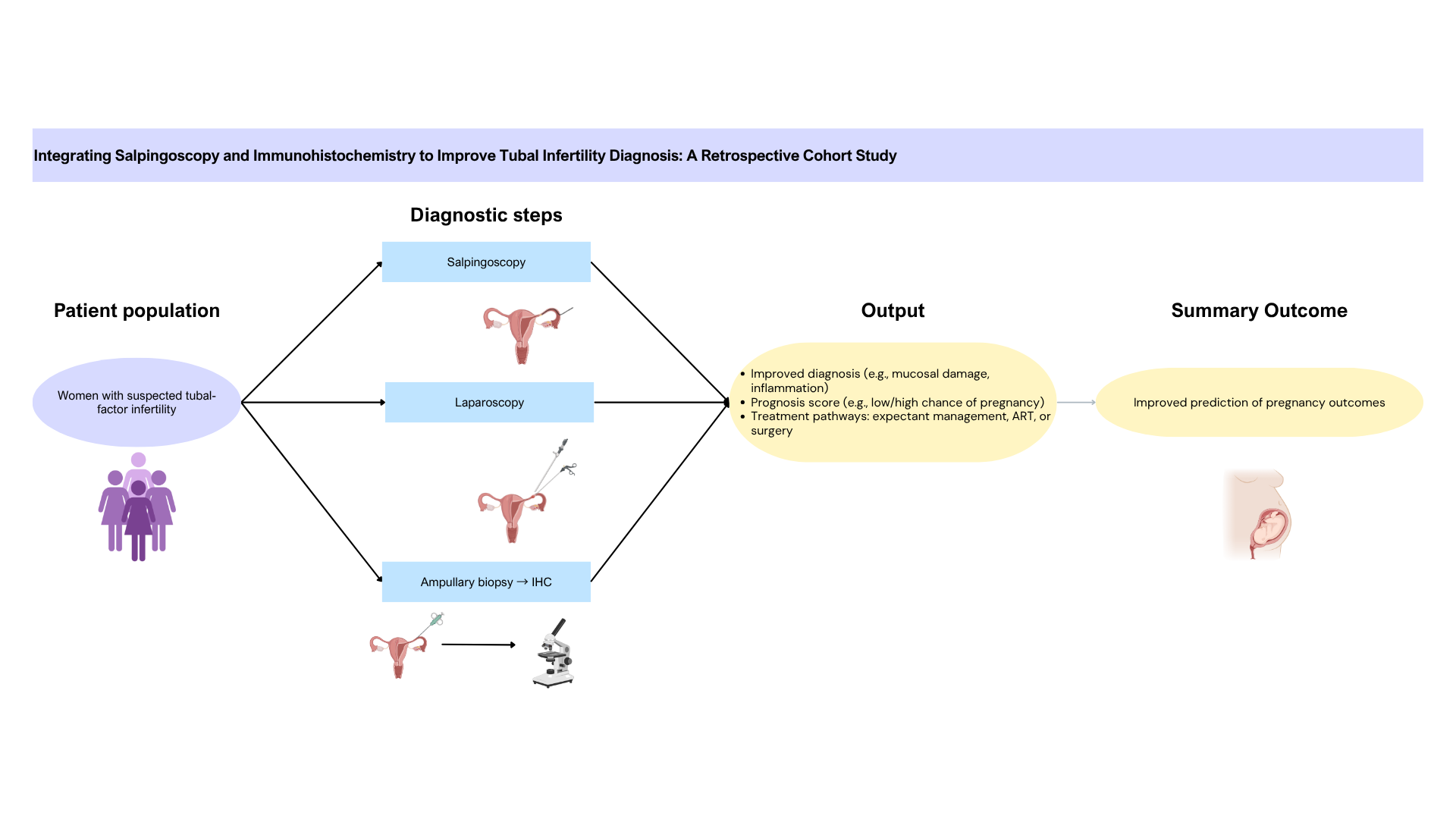

Supplement: Supplementary file 1 [file Image1.png]

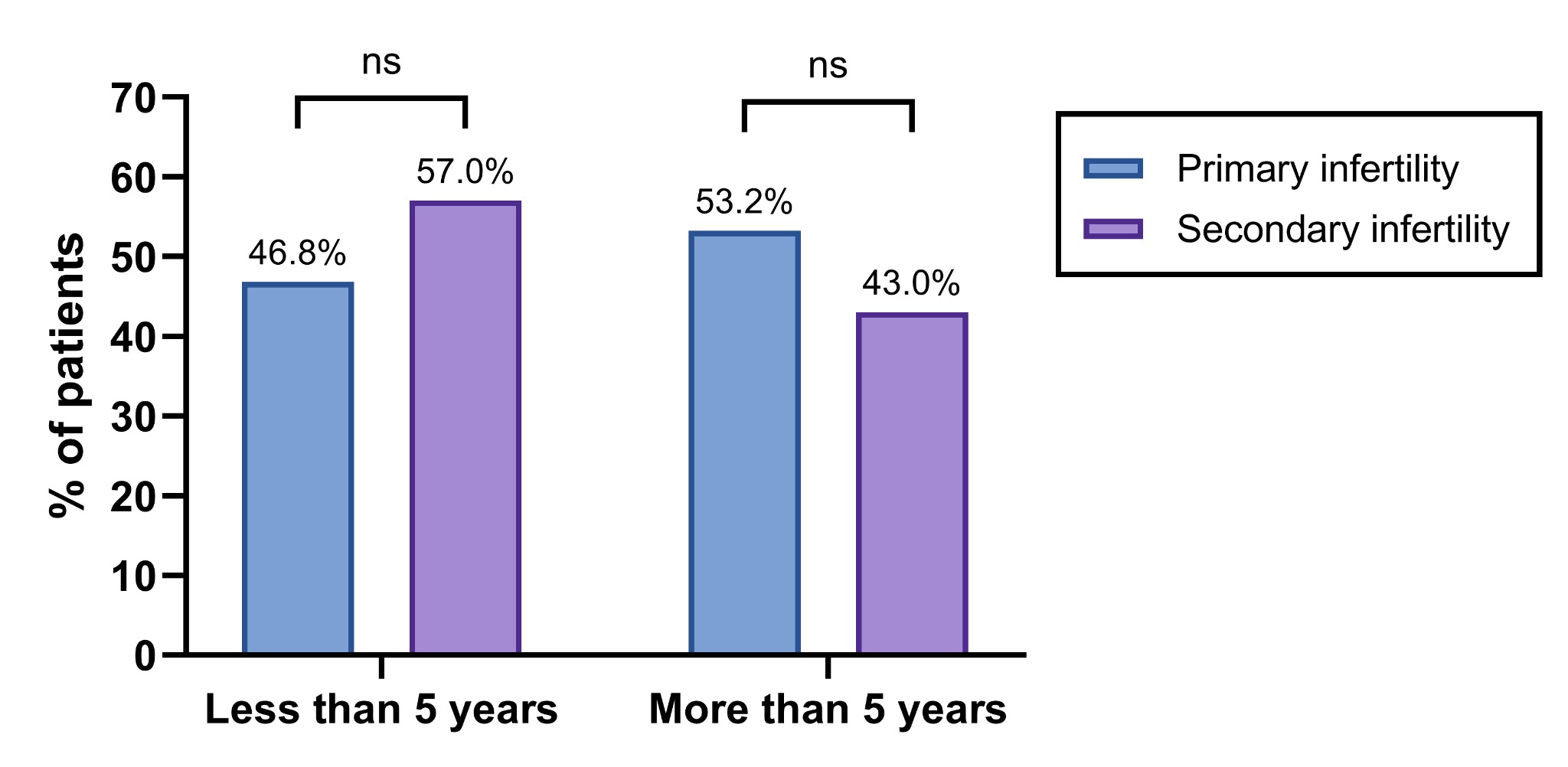

Supplement: Supplementary file 2 [file Image2.jpeg]
